# Notes and News

**Published:** 1899-05-27

**Authors:** 


### Notes and News.

(Collected and Communicated.)

### HOSPITAL MEETINGS.

National Hospital for the Paralysed and Epileptic, Queen Square.—Festival Dinner at seven p.m., on Wednesday, May 31st, at the Hotel Metropole, Right Hon. Sir Charles Hall, K.C.M.G., Q.C., M.P., in the chair.

Victoria Hospital for Children.—Festival Dinner on Friday, June 2nd, at the Hotel Cecil, Lord Roberts in the chair.

Hospital for Sick Children.—Annual Court at five p.m., on Wednesday, May 31st.

Mr. Stroyan, who has just died at Johannesburg, has bequeathed £2,000 to the Norwich Hospital and £10,000 to the Johannesburg Hospital.

Lord Overtoun has opened the new "Dr. Little Hospital" at Dumbarton, the gift of Mrs. Little to the locality.

The Middlesex Hospital has received a donation of £315 from Miss M. Dresden to endow a bed, during her life, to her father's memory.

The late Mr. Vincent Stuckey Lean has bequeathed £1,000 each to King's College Hospital, the Bristol General Hospital, the Bristol Hospital for Children, and to the Weston-super-Mare Sanatorium.

Mr. E. P. Wills has given a further £5,000 in addition to his previous donation of £20,000 towards the establishment of the Bristol Jubilee Convalescent Home.

A hospital ship has just been built at Renfrew for the Japanese Red Cross Society. She is 314 ft. long by 39 ft., with a speed of 15 knots an hour. Accommodation is provided for 300 patients, and for the necessary staff of doctors and nurses. In times of peace the ship will be utilised in the merchant service.

It is intended to erect a new hospital for women at Leeds. The existing institution which serves the purpose is too small, and it is utilised as a children's hospital also. It will be retained as such when the new hospital is provided in the same grounds, as the site is quite large enough. The women's hospital will contain three wards with eighteen beds each, and four single wards, and an operating theatre. Besides these there will be a separate maternity department containing four wards of four beds, isolating accommodation and the necessary offices. An out-patient department will also be provided. £8,000 towards the £40,000 estimated as necessary for the scheme has been provided. It is expected the whole amount will be contributed in the neighbourhood before long, and in the meanwhile the work will proceed by degrees.

T.R.H. the Duke and Duchess of York will on Thursday, June 22nd, visit Chalfont St. Peter, to open four new homes at the colony established there by the National Society for the Employment of Epileptics, of which His Royal Highness is president. These homes will, in the aggregate, increase the existing accommodation of the colony by nearly a hundred beds. The cost of building the new homes has been defrayed by special donations given respectively by Mr. Passmore Edwards, Mr. Frederick Greene, and Mrs. Dearmer, but the cost of the necessary furniture and equipment has as yet been only partially provided.

Under the title, "Echoes of Many Voices," and in connection with the forthcoming festival (Sir Charles Hall in the chair), the board of management of the National Hospital for the Paralysed and Epileptic are issuing a pamphlet, which is probably unique in its structure, every sentence having been "taken from the lips of eminent men who have publicly advocated the cause of the charity." Another distinctly novel feature is a scheme to return to donors "unable to sacrifice present income, but desirous to benefit the charity" annuities calculated at from 4 to 5½ per cent. upon the amount of the gift. Any sum from £50 upwards will be received upon these terms, and a gift of £1,000 will be considered to provide for the future endowment of a bed, upon which the donor may at once bestow a name. Absolute security for payment of the annuities is provided, and the scheme may be commended to the charitable as a sound and practicable means of perpetuating and extending the usefulness of an institution truly national in its scope. Instead of themselves receiving the annuity, donors may nominate some other person as the recipient, and in this way provision may be made for dependents and others during their lives, with an ultimate reversion of a substantial sum to the hospital. Full information will be afforded by the director at the hospital.

The Scotch Bill for the enforcement of a provision of seats for shop assistants has already passed the House of Commons without exciting much comment, but when the Bill came before the House of Lords for a second reading Lord Salisbury attacked it with much vigour, and the motion was lost. Lord Salisbury's main contention was that the matter was rather one to be left to the humane feelings of employers of labour than one for legislation. We quite agree that the matter is one which should not be dependent upon legislative interference; but the fact is that humane feelings have so far done little to secure a most reasonable provision for the comfort and health of those who serve in shops. Public opinion is gradually enforcing more and more consideration from the employer to the employed, and it is extraordinary that so obvious a reproach has been allowed to attach itself to shopkeepers for so long. This is pre-eminently a woman's question, and it seems most inconsistent that concerted action is being taken by women in political and other matters less directly affecting the sex, whilst the utmost apathy has been shown respecting this. It only needs a well-directed and influentially-signed protest to the principal shopkeepers, followed by a boycott where the appeal is disregarded, to effect the necessary reform. No one can justify the system whereby young women are forced to remain standing for seven or eight hours during five days of the week, when, without inconvenience or neglect of duty, they could find time to rest during brief intervals of leisure.

